# Polymorphisms of the *ASIP* gene and the haplotype are associated with fat deposition traits and fatty acid composition in Chinese Simmental steers

**DOI:** 10.5194/aab-62-135-2019

**Published:** 2019-03-29

**Authors:** Yinuo Liu, Xibi Fang, Zhihui Zhao, Junya Li, Elke Albrecht, Lisa Schering, Steffen Maak, Runjun Yang

**Affiliations:** 1 College of Animal Science, Jilin University, Changchun, 130062, P. R. China; 2 College of Agriculture, Guangdong Ocean University, Zhanjiang, 523088, P. R. China; 3 Institute of Animal Sciences, Chinese Academy of Agricultural Sciences, Beijing, 100193, P. R. China; 4 Institute of Muscle Biology and Growth, Leibniz Institute for Farm Animal Biology (FBN), Dummerstorf, 18196, Germany

## Abstract

Unlike specific expression in the skin of wild mice, the agouti signaling protein (ASIP) is expressed widely in the tissue of cattle, including adipose and muscle tissue. Hence, it has been suggested that ASIP plays a role in bovine fat metabolism. An inserted L1-BT element was recently identified upstream of the *ASIP* locus which led to an ectopic expression of *ASIP* mRNA in cattle. In this study, we detected the indel of the L1-BT element at g. -14643 nt and three SNPs in introns of the *ASIP* gene (g. -568A > G, g. -554A > T, and g. 4805A > T) in a Chinese Simmental steer population. The association analysis between variants of *ASIP* and economic traits showed that the homozygous genotype of L1-BT element insertion, AA genotype of g. -568A > G, and AT genotype of g. 4805A > T were significantly correlated with carcass and fat-related traits, such as live weight and back fat thickness. Moreover, three haplotypes (H1: AT; H2: AA; H3: GT) were identified by linkage disequilibrium analysis and formed six combined genotypes. Results indicated that Chinese Simmental steers with an H1H2 combined genotype had a higher measured value of fat-deposition-related traits (p<0.05), including thickness of back fat and percentage of carcass fat coverage, but a lower content of linoleic acid and α-linolenic acid (p<0.05). Individuals of an H3H3 combination had a lower marbling score, perirenal fat weight, and carcass weight (p<0.05). This suggests that these three SNPs and two combined haplotypes might be molecular markers for beef cattle breeding selection.

## 1 Introduction

The Agouti signaling protein (ASIP) has been described as a secreted protein expressed mainly in the skin and regulated pigmentation in wild mice (Bultman et al., 1992; Voisey and van Daal, 2002). Mutations in *ASIP*, such as lethal yellow ($A^{y}$) and viable yellow ($A^{\mathrm{vy}/a}$), can lead to an overexpression in various tissues, including adipose and muscle tissue (Bultman et al., 1992; Morgan et al., 1999). The ectopic *ASIP* expression in mice caused not only yellow fur but also obesity (Klebig et al., 1995). Therefore, another biological function of *ASIP* was suggested in the regulation of obesity in mice.

In human and farm animals, *ASIP* is ubiquitously expressed. *ASIP* mRNA could be detected in human adipose tissue, skin, heart, testis, ovary, liver, and kidney (Wilson et al., 1995). In pigs, the expression of *ASIP* could be found in liver and muscle (Zhao et al., 2015). Norris and Whan (2008) detected *ASIP* mRNA in the liver, kidney, skin, heart, and spleen of sheep. Several bovine studies had reported on *ASIP* mRNA expression in adipose tissue, M. longissimus, skin, heart, testis, ovary, and kidney (Girardot et al., 2005; Albrecht et al., 2012; Liu et al., 2018). Studies on *ASIP* in farm animals mainly focused on coat color (Kim et al., 2004; Norris and Whan, 2008; Mao et al., 2010; Li et al., 2014; Han et al., 2015; Zhang et al., 2017). Both Girardot et al. (2005) and Royo et al. (2005) found that there was no variant in coding exons of *ASIP* in different breeds of cattle with different coat color. A full-length element (L1-BT element; line 1) inserted at 5${}^{\prime }$ upstream of the *ASIP* gene was identified to give rise to an overexpression of *ASIP* in the skin of Normande cattle (Girardot et al., 2006). Girardot et al. (2006) concluded that the insertion of the L1-BT element may be the reason for the brindle coat color of the Normande cattle. The authors also presumed that the overexpression of *ASIP* caused by L1-BT element insertion may influence the meat production in cattle, as mice with ectopic expression of ASIP were obese. Liu et al. (2018) reported that in bulls of an $F_{2}$ generation (Charolais × Holstein) an extremely high level of *ASIP* mRNA was observed due to the L1-BT element insertion at *ASIP* in 17 bulls, but no association with fat-related traits was found. However, there were significant correlations between the *ASIP* mRNA level of subcutaneous fat and traits related to fat deposition in bulls without an L1-BT element (Liu et al., 2018). Up to now, there is no research yet for L1-BT element insertion at the *ASIP* locus in Chinese cattle populations. Also, association analyses between single nucleotide polymorphism (SNP) of bovine *ASIP* and carcass traits and fatty acid composition in cattle have not been reported. Therefore, in this study we scanned for the L1-BT element and further SNPs at the *ASIP* locus in a population consisting of 363 Chinese Simmental steers. Here we focused on the traits related to fat deposition (e.g., marbling score, back fat thickness) and fatty acid composition, which can provide important information for meat production and quality in cattle. Carcass traits like liver weight and thigh meat thickness were also analyzed, as ASIP, a secreted protein, may circulate and act in the different tissues and parts of the body. The correlation between SNPs and traits was determined to illustrate the potential effect of ASIP in cattle, especially for meat production and meat quality.

## 2 Materials and methods

### 2.1 Animals and sampling

There were 363 Chinese Simmental steers involved in this study. Animals were randomly selected from 15 cattle farms in the Wulagai administrative district of Inner Mongolia in China. Steers were slaughtered at 28 months of age at the Inner Mongolia Baolongshan cattle farm (Tongliao, China). Carcass composition traits, meat quality traits, and fatty acid composition traits were measured at the Chinese Academy of Agricultural Sciences meat laboratory as described previously by Fang et al. (2017). Fatty acid content was extracted from M. longissimus and expressed as grams per 100 g fresh tissue. Blood samples (10 mL per animal) were collected from the jugular vein with anticoagulant (acid citrate dextrose, ACD) and stored at -80 ${}^{\circ }$C until further analysis. All animal experiments in this study abided strictly by the ordinance for the care and use of laboratory animals of the Jilin University Animal Care and Use Committee (permit number: SYXK (Ji) pzpx20181227083).

### 2.2 *ASIP* variant detection and genotyping by sequencing

DNA was extracted from leucocytes from whole blood samples (10 mL from each steer) using a TIANamp Blood DNA Kit (Tiangen, Beijing, China) and following the manufacturer's protocol. The purity and concentration of the genomic DNA were determined using a NanoDrop ND-2000 ultraviolet spectrophotometer (Thermo Fisher Scientific Inc., USA), and the quality was verified by agarose gel electrophoresis. Primers (Table 1) were designed using Primer 3web (Version 4.0.0, http://primer3.ut.ee/, last access: 10 December 2018) and synthesized by GENEWIZ, Inc. (Suzhou, China). Primer pairs of the *ASIP* L1-BT element and *ASIP* 5${}^{\prime }$ UTR (untranslated region) upstream (Table 1) were used for genotyping the indel of the L1-BT element upstream of 5${}^{\prime }$ UTR of *ASIP*. Both primer pairs shared the same forward primer which was located upstream of L1-BT element insertion. The reverse primer of the *ASIP* L1-BT element was designed at genomic $5^{\prime }$ junctions of the L1-BT element, whereas the location of the *ASIP* 5${}^{\prime }$ UTR upstream reverse primer is in a genomic region without L1-BT element insertion. Standard polymerase chain reaction (PCR) for both the *ASIP* L1-BT element and the *ASIP* 5${}^{\prime }$ UTR upstream primer pairs was performed in each sample. All target sequences were amplified in a 30 µL reaction volume including 15 µL of 2 × Green Taq PCR Mix (Vazyme, Nanjing, China), 0.2 µL of forward and reverse primers (10 µM), 1 µL of template DNA (50 ng µL${}^{-1}$), and 6.1 µL of nuclease-free $H_{2}O$. The amplification started with an initial denaturation at 95 ${}^{\circ }$C for 5 min, followed by 35 cycles with 95 ${}^{\circ }$C for 30 s, a template-specific annealing temperature for 30 s, 72 ${}^{\circ }$C for 45 s, and a final step with 72 ${}^{\circ }$C for 10 min. The annealing temperatures are provided in Table 1. The PCR products of the *ASIP* L1-BT element and *ASIP* 5${}^{\prime }$ UTR upstream were run on 1 % agarose gels and visualized under UV light (Alpha Innotech, USA) to genotype the L1-BT element indel. LL (homozygote of L1-BT element insertion) and GG (homozygote of the genomic sequence without L1-BT element insertion) genotypes were identified, respectively, when only the PCR product of the *ASIP* L1-BT element and the *ASIP* 5${}^{\prime }$ UTR upstream primer pairs was observed in one sample. The heterozygote of the L1-BT element indel (GL genotype) was determined when both PCR products of the *ASIP* L1-BT element and *ASIP* 5${}^{\prime }$ UTR upstream were detected in one sample. Furthermore, PCR products were sent to GENEWIZ, Inc. (Suzhou, China), for sequencing. Sequences were analyzed by Chromas (Technelysium, Australia) to determine the SNP locations and genotypes.

**Table 1:** Primer sequences.

Primer name	Sequence (5${}^{\prime }$ → 3${}^{\prime }$)	Genbank acc. no.	Amplicon	$T_{a}$
		position	length (bp)	(${}^{\circ }$C)
*ASIP* L1-BT element${}^{1}$		NC_037340.1${}^{2}$		
For${}^{3}$	AAATCAACATCTCGGCTTGG	1–20	420	62
Rev${}^{4}$	CTTTTCTGGGTGCCTGATGT	420–398		
*ASIP* 5${}^{\prime }$ UTR upstream${}^{1}$		NC_037340.1${}^{2}$		
For	AAATCAACATCTCGGCTTGG	1–20	430	62
Rev	AAAAGGAAAGTGCGGAGGAG	8841–8822		
*ASIP*_SNP1		NC_037340.1${}^{2}$		
For	TGAGTCACTTCAGTCGTGTCT	22 306–22 326	996	58
Rev	AGCAAGGTAGCCAGGAGGA	23 301–23 263		
*ASIP*_SNP2		NC_037340.1${}^{2}$		
For	GGCTAGACTCCGAACCTACC	27 929–27 945	783	59
Rev	GCTAAGTAACCGCGTTTCCC	28 711–28 692		

${}^{1}$ Adopted from Albrecht et al. (2012). ${}^{2}$ Nucleotide positions refer to partial sequence of NC_037340.1 starting at nt 63 639 542 (i.e., nt 1 in this table is nt 63 639 542). ${}^{3}$ For: forward primer. ${}^{4}$ Rev: reverse primer.

### 2.3 Statistical analysis

The genotype frequency and allelic frequency of each SNP were calculated according to genotyping results. The polymorphism information component (PIC) was determined by Botstein's methods (Botstein et al., 1980). The Hardy–Weinberg equilibrium of the polymorphisms was tested with the chi-squared ($\chi^{2}$) test. Associations between *ASIP* gene polymorphisms and economic traits of beef cattle were analyzed by two-way analysis of variance (ANOVA) using SPSS 13.0 for Windows. The fixed model was

1 $$Y_{ijk}=u+ys_{i}+m_{j}+e_{ijk},$$

where $Y_{ijk}$ is the observed value of the kth individual from the Simmental breed of genotype j in the ith-year season, u is the lowest square mean of the observed values, $ys_{i}$ is the effective value of the ith-year season, $m_{j}$ is the effective value of genotype j, and $e_{ijk}$ is the random residual effect corresponding to the observed value. For SNPs that correlated with the carcass and fat-related traits, the haplotype test and linkage disequilibrium (LD) were performed and measured by $D^{\prime }$ and $r^{2}$ with the HaploView software (Daly Lab at the Broad Institute Cambridge, USA, ver. 3.32) (Barrett et al., 2005).

## 3 Results

### 3.1 Screening of SNP loci of the *ASIP* gene in Chinese Simmental population

**Figure 1:**
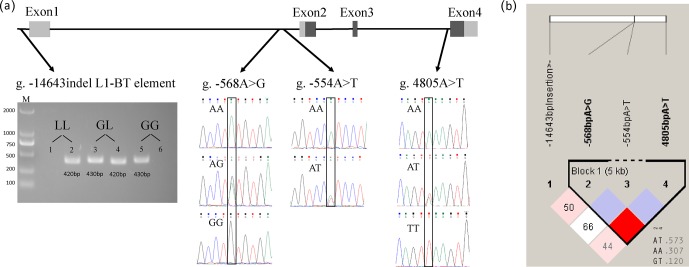
SNP detection at the *ASIP* locus in Chinese Simmental steers and linkage and haplotype analysis. **(a)** Non-coding (gray) and coding (black) exons are presented as a box. For the g. -14643indel L1-BT element, PCR products in lane 1 and 2, lane 3 and 4, and lane 5 and 6 were amplified from templates of three different steers. The LL genotype (homozygote of L1-BT element insertion) was presented when only the PCR product of the *ASIP* L1-BT element primer pair was detected in one sample. The GL genotype (heterozygote of L1-BT element insertion) was determined when PCR products were amplified by both primer pairs of *ASIP* 5${}^{\prime }$ UTR upstream and the *ASIP* L1-BT element in one sample. The GG genotype (no L1-BT element inserted) was identified when only the PCR product of the *ASIP* 5${}^{\prime }$ UTR upstream primer pair was detected in one sample. Lanes: M – size marker; 1, 3, 5 – specific PCR products of *ASIP* 5${}^{\prime }$ UTR upstream primer pair at 430 bp are genomic sequences with no L1-BT element; 2, 4, 6 – PCR products (420 bp) containing the partial sequence of the L1-BT element. **(b)** Box with red color presents strong linkage between g. -568A > G and g. 4805A > T. Three haplotypes are shown with haplotype frequency.

Sequencing results showed that all PCR products are specific and correct as designed. The indel of the L1-BT element upstream of 5${}^{\prime }$ UTR of *ASIP* (at g. -14643 nt) was detected by electrophoresis (Fig. 1a). Three SNPs, including g. -568A > G (located in intron1), g. -554A > T (located in intron1), and g. 4805A > T (located in intron3), were identified in 363 Chinese Simmental steers and genotyped according to sequencing results (Fig. 1a).

### 3.2 Genetic diversity of SNPs in the *ASIP* gene in Chinese Simmental population

Values of genotypic frequencies, allelic frequencies, PIC, Ho (gene homozygosity), and He (gene heterozygosity) were calculated to illustrate the genetic diversity of *ASIP* in Chinese Simmental steers (Table 2). The percentage of Ho in three SNPs was higher than that of He. The PIC value of g. -568A > G, 0.29, indicated an intermediate polymorphism frequency of SNP (0.25 < PIC value < 0.5, intermediate polymorphism). g. -554A > T with a 0.12 PIC value showed a low polymorphism frequency (PIC value < 0.25, low polymorphism). g. 4805A > T had a high polymorphism frequency with a 0.5 PIC value (PIC value > 0.5, high polymorphism). All three SNPs fit the Hardy–Weinberg equilibrium in the population (P>0.05).

**Table 2:** Genetic diversity of the *ASIP* gene.

		Allele									
Variants${}^{*}$	Location	frequency			Genotype frequency			PIC${}^{a}$	Ho${}^{b}$	He${}^{c}$	Variant ID
L1-BT element indel${}^{d}$	Upstream	L	G		LL	GL	GG	–	–	–	–
		0.146	0.854		0.006	0.281	0.713				
g. -568A > G	Intron1	A	G		AA	AG	CC	0.29	0.80	0.20	rs379766904
		0.888	0.112		0.787	0.201	0.012				
g. -554A > T	Intron1	A	T		AA	AT	TT	0.12	0.93	0.07	rs382441624
		0.965	0.035		0.930	0.070	0.00				
g. 4805A > T	Intron3	A	T		AA	TA	TT	0.50	0.61	0.39	rs208055105
		0.306	0.694		0.110	0.392	0.498				

${}^{*}$ The A base of the start codon ATG was regarded as the first base. ${}^{a}$ Polymorphism information component. ${}^{b}$ Ho: gene homozygosity. ${}^{c}$ He: gene heterozygosity. ${}^{d}$ LL: homozygote of L1-BT element insertion; GL: heterozygote of L1-BT element insertion; GG: no L1-BT element inserted.

### 3.3 Correlation analyses of *ASIP* polymorphisms with carcass and fat deposition traits

The associations between variants and traits are presented in Table 3. Steers heterozygous of the L1-BT element indel had a heavier liver weight (P<0.05). Compared to other two genotypes, individuals carrying a homozygous insertion of the L1-BT element possessed a lower value regarding the weight of the kidneys (P<0.05) and perirenal fat (P<0.01), in the thickness of waist meat (P<0.05) and back fat (P<0.01), and in the percentage of carcass fat coverage (P<0.01). For g. -568 nt, steers of the AA genotype had a higher live weight (P<0.05) and carcass pH values (P<0.05) than GG genotype individuals. Moreover, the marbling score of AA and AT genotype steers was higher than in GG steers (P<0.05). At g. 4805 nt of *ASIP*, a heavier liver weight (P<0.01), carcass weight (P<0.05), and higher dressing percentage (P<0.05) were observed in individuals with the AT genotype compared with the TT genotype. Steers of the AT genotype had thicker back fat and a higher carcass fat coverage rate. There was no significant association between g. -554A > T and the investigated traits. In fatty acid composition, there was no statistical difference among genotypes of each SNP in either saturated fatty acid content or in polyunsaturated fatty acid content (data not shown).

**Table 3:** Correlation analyses of *ASIP* polymorphisms with carcass and fat deposition traits in Chinese Simmental steers.

Variants	Genotypes	LW	CW	Carcass	DP	Liver	Kidney	PFW	TMT	WMT	BFT	FCR	MBS
		(kg)	(kg)	PH	(%)	(kg)	(kg)	(kg)	(cm)	(cm)	(cm)	(%)	
		Mean ± SD	Mean ± SD	Mean ± SD	Mean ± SD	Mean ± SD	Mean ± SD	Mean ± SD	Mean ± SD	Mean ± SD	Mean ± SD	Mean ± SD	Mean ± SD
L1-BT	LL	443.50±25.46	228.10±6.36	6.65±0.07	51.48±1.52	$4.94\pm 1.03^{a}$	$0.91\pm 0.04^{a}$	$1.23\pm 0.69^{A}$	$17.40\pm 1.27^{\mathrm{ab}}$	$5.65\pm 0.21^{a}$	$0.20\pm 0.00^{A}$	$17.50\pm 3.53^{A}$	6.00±0.00
element	GL	493.99±56.46	258.32±36.23	6.26±0.50	52.16±2.07	$5.97\pm 1.06^{b}$	$1.16\pm 0.18^{b}$	$4.75\pm 2.90^{B}$	$18.27\pm 1.58^{a}$	$6.89\pm 0.91^{b}$	$0.95\pm 0.64^{B}$	$48.35\pm 20.30^{B}$	5.33±0.69
indel	GG	488.60±63.58	255.9±40.36	6.30±0.52	52.06±2.48	$5.79\pm 1.12^{a}$	$1.16\pm 0.21^{b}$	$4.43\pm 2.75^{B}$	$17.81\pm 1.70^{b}$	$6.79\pm 0.90^{b}$	$0.92\pm 0.61^{B}$	$47.35\pm 22.29^{B}$	5.41±0.72
g. -568A > G	AA	$491.56\pm 61.38^{a}$	256.98±38.7	$6.32\pm 0.52^{a}$	52.14±2.20	5.82±1.12	1.16±0.20	4.50±2.80	17.97±1.65	6.80±0.92	0.90±0.61	47.32±22.31	$5.40\pm 0.70^{a}$
	AG	$485.94\pm 58.48^{\mathrm{ab}}$	252.73±38.02	$6.17\pm 0.51^{b}$	51.88±2.89	5.95±1.09	1.17±0.21	4.49±2.75	17.72±1.82	6.79±0.84	0.98±0.67	47.33±20.64	$5.43\pm 0.70^{a}$
	GG	$433.50\pm 80.07^{b}$	226.78±45.14	$5.95\pm 0.51^{b}$	52.25±1.78	5.53±1.07	1.03±0.24	3.02±1.67	18.15±1.42	7.08±1.20	1.05±0.66	43.50±23.29	$4.75\pm 0.96^{b}$
g. -554A > T	AA	488.16±61.35	255.03±38.88	6.30±0.52	52.10±2.37	5.79±1.1	1.15±0.21	4.41±2.75	17.93±1.66	6.79±0.90	0.91±0.62	46.83±21.79	5.43±0.69
	AT	510.96±55.64	265.73±35.85	6.14±0.50	51.88±1.93	6.45±1.09	1.24±0.17	5.46±3.08	17.84±1.95	6.94±0.91	1.07±0.65	53.25±23.38	4.96±0.81
g. 4805A > T	AA	500.33±62.50	$261.25\pm 60.74^{\mathrm{ab}}$	6.24±0.45	$52.12\pm 1.81^{\mathrm{ab}}$	$5.81\pm 1.06^{\mathrm{AB}}$	1.12±0.19	4.27±2.97	18.20±2.05	$6.82\pm 0.83^{\mathrm{ab}}$	$0.85\pm 0.66^{b}$	$46.4\pm 22.55^{a}$	5.34±0.80
	AT	497.28±53.35	$261.39\pm 35.75^{a}$	6.26±0.52	$52.43\pm 2.24^{a}$	$6.02\pm 1.05^{A}$	1.17±0.18	4.79±2.66	18.02±1.62	$6.94\pm 0.91^{a}$	$1.03\pm 0.64^{a}$	$50.19\pm 21.47^{b}$	5.38±0.67
	TT	485.93±65.40	$252.38\pm 40.79^{b}$	6.29±0.54	$51.80\pm 2.62^{b}$	$5.77\pm 1.16^{B}$	1.17±0.23	4.50±2.80	17.83±1.64	$6.76\pm 0.91^{b}$	$0.91\pm 0.62^{b}$	$62.24\pm 9.74^{a}$	5.40±0.71

Note: mean trait values marked with different lowercase letters in the same column are significantly different (P<0.05); mean trait values marked different uppercase letters in the same column are extremely significantly different (P<0.01). LW: live weight; CW: carcass weight; DP: dressing percentage; PFW: perirenal fat weight; TMT: thigh meat thickness; WMT: waist meat thickness; BFT: back fat thickness; FCR: carcass fat coverage rate; MBS: marbling score.

### 3.4 Haplotype analysis and association analyses of dominant haplotypes with carcass traits and fatty acid composition

The linkage analysis of three investigated SNPs in *ASIP* is shown in Fig. 1b. Linkage was observed only between SNPs at g. -568 nt and g. 4805 nt ($D^{\prime }=1$; LOD (logarithm of the odds) = 6.7; $r^{2}=0.06$). Three haplotypes (H1: AT; H2: AA; H3: GT) were detected and generated six haplotype combinations. The combined haplotypes H1H2 and H3H3 had the highest and lowest genotypic frequency: 33.5 % and 1.3 %, respectively. The correlation analysis (Table 4) indicated that steers with an H1H2 genotype had higher live weight, carcass weight, and perirenal fat than those with an H3H3 haplotype combination (P<0.05). The thickness of back fat was significantly greater in steers with an H1H2 combination compared to that with H1H1 (P<0.01) and H2H2 (P<0.05). A higher carcass fat coverage rate could be observed in H1H2 genotype individuals compared to H1H1 (P<0.01) and H2H2 steers (P<0.05). The carcass pH value of H1H2 steers was significantly higher than that of H1H3 (P<0.05) and H3H3 (P<0.05) individuals. Moreover, individuals with an H3H3 haplotype combination got a lower marbling score than steers of H1H1, H1H2, or H1H3 (P<0.05).

**Table 4:** The effect of six haplotype combinations on carcass and fat deposition traits in Chinese Simmental steers.

Genotypes${}^{*}$	LW	CW	Carcass	DP	Liver	Kidney	PFW	GFW	BFT	FCR	MBS
	(kg)	(kg)	PH	(%)	(kg)	(kg)	(kg)	(kg)	(cm)	(%)	
	Mean ± SD	Mean ± SD	Mean ± SD	Mean ± SD	Mean ± SD	Mean ± SD	Mean ± SD	Mean ± SD	Mean ± SD	Mean ± SD	Mean ± SD
H1H1	$488.75\pm 66.33^{\mathrm{ab}}$	$254.46\pm 41.69^{\mathrm{ab}}$	$6.36\pm 0.55^{a}$	$51.90\pm 2.46^{\mathrm{ab}}$	$5.70\pm 1.18^{a}$	$1.16\pm 0.23^{\mathrm{ab}}$	$4.41\pm 2.78^{\mathrm{ab}}$	$0.93\pm 0.32^{a}$	$0.86\pm 0.60^{b}$	$45.28\pm 22.88^{b}$	$5.43\pm 0.68^{a}$
H1H2	$498.29\pm 54.77^{a}$	$261.87\pm 35.99^{a}$	$6.29\pm 0.51^{\mathrm{ac}}$	$52.42\pm 2.12^{a}$	$5.99\pm 1.11^{\mathrm{bc}}$	$1.18\pm 0.18^{\mathrm{ab}}$	$4.76\pm 2.68^{a}$	$0.89\pm 0.34^{\mathrm{ab}}$	$1.00\pm 0.63^{a}$	$50.64\pm 22.01^{a}$	$5.38\pm 0.67^{a}$
H1H3	$484.00\pm 63.35^{\mathrm{ab}}$	$250.55\pm 39.39^{\mathrm{ab}}$	$6.15\pm 0.51^{\mathrm{bd}}$	$51.65\pm 2.98^{b}$	$5.99\pm 1.18^{\mathrm{bc}}$	$1.19\pm 0.23^{a}$	$4.51\pm 2.77^{\mathrm{ab}}$	$0.81\pm 0.33^{b}$	$0.97\pm 0.65^{\mathrm{ab}}$	$48.70\pm 21.16^{\mathrm{ac}}$	$5.43\pm 0.72^{a}$
H2H2	$499.28\pm 63.12^{a}$	$260.46\pm 38.05^{\mathrm{ab}}$	$6.26\pm 0.44^{\mathrm{ad}}$	$52.07\pm 1.82^{\mathrm{ab}}$	$5.81\pm 1.07^{\mathrm{ac}}$	$1.12\pm 0.19^{b}$	$4.23\pm 3.00^{\mathrm{ab}}$	$0.98\pm 0.37^{a}$	$0.84\pm 0.67^{b}$	$46.00\pm 22.76^{\mathrm{bc}}$	$5.32\pm 0.81^{\mathrm{ab}}$
H2H3	$492.55\pm 47.72^{\mathrm{ab}}$	$259.06\pm 36.21^{\mathrm{ab}}$	$6.16\pm 0.51^{\mathrm{bcd}}$	$52.42\pm 2.87^{\mathrm{ab}}$	$6.12\pm 0.64^{\mathrm{bc}}$	$1.16\pm 0.14^{\mathrm{ab}}$	$4.93\pm 2.69^{a}$	$0.99\pm 0.36^{a}$	$1.14\pm 0.68^{a}$	$47.80\pm 18.89^{\mathrm{ab}}$	$5.35\pm 0.67^{\mathrm{ab}}$
H3H3	$433.50\pm 80.07^{b}$	$226.78\pm 45.14^{b}$	$5.95\pm 0.51^{\mathrm{bd}}$	$52.25\pm 1.78^{\mathrm{ab}}$	$5.43\pm 1.07^{\mathrm{ac}}$	$1.03\pm 0.24^{\mathrm{ab}}$	$3.02\pm 1.67^{b}$	$0.75\pm 0.23^{\mathrm{ab}}$	$1.05\pm 0.66^{\mathrm{ab}}$	$43.50\pm 23.29^{\mathrm{ab}}$	$4.75\pm 0.96^{b}$

${}^{*}$ H1: AT; H2: AA; H3: GT. Note: mean trait values marked with different letters in the same column are significantly different (P<0.05). LW: live weight; CW: carcass weight; DP: dressing percentage; PFW: perirenal fat weight; GFW: genital fat weight; BFT: back fat thickness; FCR: carcass fat coverage rate; MBS: marbling score.

The association between dominant haplotypes and the content of either saturated fatty acids or polyunsaturated fatty acids was analyzed. Results indicated that the content of linoleic acid in longissimus dorsi of H1H2 steers was lower than that in H1H3 steers (P<0.05) (Table 5). Moreover, a lower content of α-linolenic acid was observed in individuals with an H1H2 genotype compared to that in H2H2 steers (P<0.05) (Table 5).

**Table 5:** The effect of five haplotype combinations on fatty acid components in Chinese Simmental steers.

Fatty acid content (g 100 g${}^{-1}$)	Genotype${}^{*}$				
	H1H1	H1H2	H1H3	H2H2	H2H3
Myristic acid (c14:0)	0.0220±0.018	0.0223±0.020	0.0171±0.007	0.0187±0.012	0.0128±0.008
Nutmeg oleic acid (c14:1)	0.0023±0.005	0.0031±0.007	0.0019±0.003	0.0012±0.003	0.0025±0.003
Hexadecanoic acid (c16:0)	0.2701±0.183	0.2809±0.257	0.2279±0.090	0.2484±0.133	0.1979±0.082
Palmitoleic acid (c16:1)	0.0282±0.020	0.0329±0.050	0.0239±0.009	0.0216±0.011	0.0261±0.012
Margaric acid (c17:0)	0.0120±0.007	0.0114±0.008	0.0108±0.005	0.0108±0.007	0.0072±0.004
Heptadecanoic acid (c17:1)	0.0052±0.006	0.0059±0.010	0.0046±0.005	0.0046±0.005	0.0052±0.005
Stearic acid (c18:0)	0.1995±0.123	0.1879±0.118	0.1860±0.083	0.1839±0.107	0.1308±0.038
Oleic acid (C18:1n9c)	0.3619±0.237	0.4393±0.613	0.3087±0.136	0.3338±0.163	0.2877±0.126
Linoleic acid (C18:2n6c)	$0.1070\pm 0.040^{\mathrm{ab}}$	$0.0927\pm 0.025^{a}$	$0.1172\pm 0.051^{b}$	$0.1087\pm 0.033^{\mathrm{ab}}$	$0.0888\pm 0.002^{\mathrm{ab}}$
α-Linolenic acid (ac18:3n3)	$0.0068\pm 0.009^{\mathrm{ab}}$	$0.0048\pm 0.005^{a}$	$0.0069\pm 0.008^{\mathrm{ab}}$	$0.0099\pm 0.007^{b}$	$0.0039\pm 0.004^{\mathrm{ab}}$
Arachidic acid (c20:0)	0.0015±0.005	0.0004±0.001	0.0004±0.001	0.0003±0.001	0.0008±0.001
Eicosenoic an acid (c20:1)	0.0005±0.002	0.0007±0.004	0.0003±0.001	0.0002±0.001	0.0007±0.001
Dohono-c-linolenic acid (c20:3n6)	0.0101±0.003	0.0099±0.003	0.0106±0.002	0.0097±0.001	0.0100±0.002
Arachidonic acid (c20:4n6)	0.0525±0.014	0.0488±0.015	0.0506±0.011	0.0470±0.013	0.0483±0.009

${}^{*}$ H1: AT; H2: AA; H3: GT. Note: data were presented as mean ± SD. Mean trait values marked with different letters in the same row are significantly different (P<0.05).

## 4 Discussion

In mice, there are five dominant mutations causing ectopic expression of *ASIP* (Voisey and van Daal, 2002). Insertions of different sizes upstream of first coding exon are the cause for intracisternal A-particle yellow ($A^{\mathrm{iapy}}$), intermediate yellow ($A^{\mathrm{iy}}$), sienna yellow ($A^{\mathrm{sy}}$), and viable yellow ($A^{\mathrm{vy}}$) mutation (Duhl et al., 1994; Michaud et al., 1994b; Siracusa et al., 1995). In the case of the $A^{y}$ mutation, *ASIP* is controlled by the *RALY* promoter due to the deletion at both the *RALY* and the *ASIP* locus. It happened only in the $A^{y}$ mutation that the homozygosity of $A^{y}$ resulted in embryonic lethality (Michaud et al., 1994a). Long interspersed nuclear elements (lines; L1s) known as autonomously mobile DNA sequences belong to autonomous retrotransposon lacking long terminal repeats (LTRs) (Kazazian and Moran, 1998). This is one of the most abundant retrotransposon in eukaryotes (Kazazian and Moran, 1998; Walsh et al., 2013). Girardot et al. (2005) observed L1-BT element insertion upstream of *ASIP* in bulls. The insertion of the L1-BT element formed a new non-coding exon which resulted in an overexpression of *ASIP* (Girardot et al., 2005). Both the homozygote and heterozygote of the L1-BT element indel were detected in bulls of the Montbéliarde breed (Girardot et al., 2006). Albrecht et al. (2012) found heterozygote insertion of this element, which gave rise to an ectopic expression of *ASIP* in different tissues of Japanese Black and Charolais cattle. In our study, three genotypes of the L1-BT element indel were also detected. It is obvious that homozygosity of L1-BT element insertion is not a lethal factor in cattle. Liu et al. (2018) reported that steers with a heterozygote of the L1-BT element indel had an extremely high *ASIP* mRNA level in various tissues, but it was not associated with any traits related to fat deposition. In the current study, there was no significant difference between GL and GG genotype Chinese Simmental steers in fat-related traits. However, steers with the LL genotype had lower perirenal fat weight, thinner back fat, and less carcass fat coverage percentage compared with other genotypes (P<0.05). According to previous knowledge that *ASIP* is highly expressed in L1-BT-inserted bulls (Girardot et al., 2006), it can be presumed that in this Chinese Simmental population overexpression of *ASIP* in steers with the LL genotype showed a negative association with traits related to fat deposition. In a previous study, we found that a heterozygous insertion of L1-BT led to an ectopic expression of *ASIP* mRNA but not protein (Liu et al., 2018). Hence, further experiments should focus on the expression pattern and effect of *ASIP* in cattle with the LL genotype.

In this study, we detected the L1-BT element indel and three SNPs at the bovine *ASIP* locus. Among them, indel of the L1-BT element at g. -14643 nt, g. -568A > G, and g. 4805A > T showed significant associations with traits related to fat deposition (e.g., back fat thickness and carcass fat coverage rate) in 363 Chinese Simmental steers. The mutations of g. -568A > G and g. 4805A > T occurred in introns and did not alter the amino acid sequence of ASIP. However, mutations in introns may also influence the expression and function of a gene by aberrant splicing (Komar, 2007; Shastry, 2009). Coding exons were scanned for SNPs in this study, but no variant of *ASIP* could be found (data not shown). Likewise, Girardot et al. (2005) and Royo et al. (2005) did not find any SNP of bovine *ASIP* in coding exons. This suggested that the coding region of bovine *ASIP* is highly conserved. The analysis of haplotypes is an important component of genetic association analysis. A haplotype consists of a group of linked SNP markers, and it may increase the coverage value of genotype over single SNP analysis (Stram, 2017). The association analysis between haplotype combinations and traits of Chinese Simmental steers indicated that the H1H2 genotype was positively correlated to carcass and meat quality traits, e.g., with a higher carcass weight and thicker back fat. But the H3H3 genotype had a negative association with fat-related traits such as a lower marbling score and perirenal fat weight. Moreover, linoleic acid and α-linolenic acid are essential fatty acids which can only be obtained from the diet (Spector and Kim, 2015). Our results showed that H1H2 steer individuals had a lower content of linoleic acid and α-linolenic acid than the H1H3 and H2H2 genotypes, respectively. These results may illustrate that Chinese Simmental steers with an H1H2 genotype may have more fat deposition in the body but less essential fatty acids, e.g., linoleic acid and α-linolenic acid, in longissimus dorsi.

## 5 Conclusion

In summary, both polymorphisms and haplotype analysis of *ASIP* showed significant correlations with fat-related traits in Chinese Simmental steers. This suggests that ASIP is involved in fat deposition at different stages of the growth and fattening of beef cattle, such as intramuscular fat, back fat, and perirenal fat deposition. The haplotype combinations of H1H2 and H3H3 may be molecular markers for traits related to fat deposition and meat quality (e.g., fatty acid composition). However, further studies are necessary to unravel the mechanism of how the *ASIP* gene affects meat production and quality in cattle.

## Data Availability

The data sets are available upon request from the corresponding author.
